# Cognitive function in severe progressive multiple sclerosis

**DOI:** 10.1093/braincomms/fcae226

**Published:** 2024-07-02

**Authors:** Dejan Jakimovski, Robert Zivadinov, Zachary Weinstock, Alex Burnham, Taylor R Wicks, Christopher Suchan, Tommaso Sciortino, Ferdinand Schweser, Niels Bergsland, Michael G Dwyer, Svetlana P Eckert, David Young-Hong, Bianca Weinstock-Guttman, Ralph H B Benedict

**Affiliations:** Buffalo Neuroimaging Analysis Center, Department of Neurology, Jacobs School of Medicine and Biomedical Sciences, University at Buffalo, State University of New York, Buffalo, NY 14203, USA; Buffalo Neuroimaging Analysis Center, Department of Neurology, Jacobs School of Medicine and Biomedical Sciences, University at Buffalo, State University of New York, Buffalo, NY 14203, USA; Center for Biomedical Imaging at the Clinical Translational Science Institute, University at Buffalo, State University of New York, Buffalo, NY 14203, USA; Buffalo Neuroimaging Analysis Center, Department of Neurology, Jacobs School of Medicine and Biomedical Sciences, University at Buffalo, State University of New York, Buffalo, NY 14203, USA; The Boston Home, Dorchester, MA 02124, USA; Buffalo Neuroimaging Analysis Center, Department of Neurology, Jacobs School of Medicine and Biomedical Sciences, University at Buffalo, State University of New York, Buffalo, NY 14203, USA; Buffalo Neuroimaging Analysis Center, Department of Neurology, Jacobs School of Medicine and Biomedical Sciences, University at Buffalo, State University of New York, Buffalo, NY 14203, USA; Buffalo Neuroimaging Analysis Center, Department of Neurology, Jacobs School of Medicine and Biomedical Sciences, University at Buffalo, State University of New York, Buffalo, NY 14203, USA; Buffalo Neuroimaging Analysis Center, Department of Neurology, Jacobs School of Medicine and Biomedical Sciences, University at Buffalo, State University of New York, Buffalo, NY 14203, USA; Center for Biomedical Imaging at the Clinical Translational Science Institute, University at Buffalo, State University of New York, Buffalo, NY 14203, USA; Buffalo Neuroimaging Analysis Center, Department of Neurology, Jacobs School of Medicine and Biomedical Sciences, University at Buffalo, State University of New York, Buffalo, NY 14203, USA; Buffalo Neuroimaging Analysis Center, Department of Neurology, Jacobs School of Medicine and Biomedical Sciences, University at Buffalo, State University of New York, Buffalo, NY 14203, USA; Department of Neurology, Jacobs Comprehensive MS Treatment and Research Center, Jacobs School of Medicine and Biomedical Sciences, University at Buffalo, State University of New York, Buffalo, NY 14203 USA; The Boston Home, Dorchester, MA 02124, USA; Department of Neurology, Jacobs Comprehensive MS Treatment and Research Center, Jacobs School of Medicine and Biomedical Sciences, University at Buffalo, State University of New York, Buffalo, NY 14203 USA; Department of Neurology, Jacobs Comprehensive MS Treatment and Research Center, Jacobs School of Medicine and Biomedical Sciences, University at Buffalo, State University of New York, Buffalo, NY 14203 USA

**Keywords:** progressive MS, ATOPS, MRI, cortical volume, thalamic volume

## Abstract

Cognitive impairment is common in multiple sclerosis and negatively impacts quality of life. Cognitive status has yet to be described in people with severe progressive multiple sclerosis, in whom conventional neuropsychological testing is exceptionally difficult. The objective for the study was to characterize cognitive performance in severe progressive multiple sclerosis and compare them with age-, sex- and disease duration-matched less disabled people with multiple sclerosis using a specifically developed auditory, non-motor test of attention/cognitive processing speed—Auditory Test of Processing Speed. Also, we aimed to determine the relationship between cognitive performance and MRI-based outcomes in these matched cohorts. The Comprehensive Assessment of Severely Affected Multiple Sclerosis study was carried out at the University at Buffalo and the Boston Home, a skilled nursing facility in Dorchester, MA. Inclusion criteria were age 30–80 years and expanded disability status scale 3.0–6.5 for community-dwelling and 7.0–9.5 for skilled nursing facility people with multiple sclerosis. The cognitive assessment was performed using the Brief International Cognitive Assessment for Multiple Sclerosis consisting of Symbol Digit Modalities Test, Brief Visuospatial Memory Test—Revised, California Verbal Learning Test—2nd edition along with Auditory Test of Processing Speed, Paced Auditory Serial Addition Test—3 second and Controlled Oral Word Association Test. MRI scans were retrospectively collected and analysed for lesion and volumetric brain measurements. The rate of completion and performance of the cognitive tests was compared between the groups, and the relationship with MRI measures was determined using sex, age and years of education-adjusted linear regression models. Significantly greater percentage of the severe multiple sclerosis group completed Auditory Test of Processing Speed when compared with the current gold standard of Symbol Digit Modalities Test (93.2% versus 65.9%). Severe progressive multiple sclerosis had worse cognitive performance in all cognitive domains with greatest differences for cognitive processing speed (Symbol Digit Modalities Test > Paced Auditory Serial Addition Test—3 second > Auditory Test of Processing Speed, Cohen's *d* < 2.13, *P* < 0.001), learning and memory (Cohen's *d* < 1.1, *P* < 0.001) and language (Controlled Oral Word Association Test with Cohen's *d* = 0.97, *P* < 0.001). Multiple cognitive domains were significantly associated with lower thalamic (standardized β < 0.419, *P* < 0.006) and cortical (standardized β < 0.26, *P* < 0.031) volumes. Specially designed (auditory) cognitive processing speed tests may provide more sensitive screening of cognitive function in severe progressive multiple sclerosis. The cognitive profile of severe multiple sclerosis is proportional to their physical outcomes and best explained by decreased grey matter volume.

## Teaser

The study by Jakimovski *et al*. utilized the recently proposed Auditory Test of Processing Speed (ATOPS) that allowed cognitive and MRI characterization of severely disabled people with multiple sclerosis from a specialized nursing facility and demonstrated significantly worse outcomes when compared with sex-, age- and disease duration-matched community-dwelling counterparts.

## Graphical Abstract

**Graphical Abstract:**
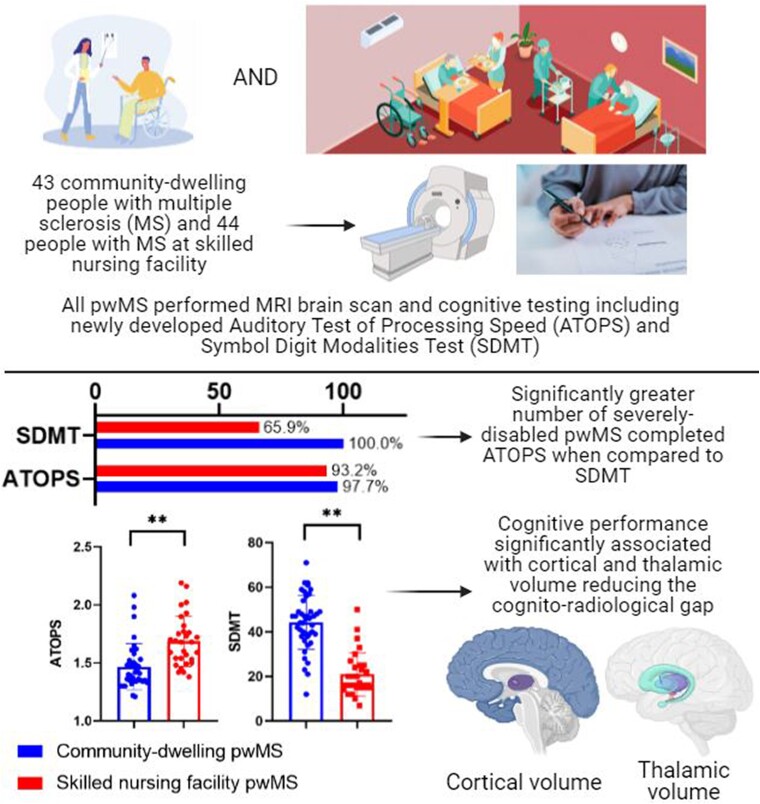

## Introduction

Multiple sclerosis is a chronic, inflammatory, neurodegenerative and demyelinating disease of the central nervous system that imposes significant physical and/or cognitive disability.^1^ In a minority of people with multiple sclerosis, the disease has an aggressive trajectory that is characterized by severe relapses with incomplete recovery and fast disability progression.^2^ A recent international forum focused on defining ‘aggressive’ multiple sclerosis emphasized the lack of well-designed studies that could provide greater clarity about this particular phenotype.^3^ Generally, the aggressive phenotype is either described as fast disability progression within a short period of time [expanded disability status scale (EDSS) of ≥6.0 within 10 years of disease onset) or with severe inflammatory activity without response to therapy (≥2 clinical relapses or MRI activity with incomplete resolution).^3^ Additionally, the panel concluded that extensive widespread neuroaxonal damage and loss (i.e. neurodegeneration) may be viewed as the neuropathologic counterpart to aggressive multiple sclerosis and drive the sustained disability progression.^3,4^ Another path to severe multiple sclerosis disability is chronic accumulation of deficits over time stemming from long-standing multiple sclerosis–induced pathology. These long-term disability trajectories are also accompanied by cognitive worsening that accrues over time. In some instances, the multiple sclerosis cognitive disability accrual can be related to the inflammatory activity during the relapsing stage and further accelerates through the progressive (neurodegenerative) phase of the disease.^5^ Recent evidence also suggests that progression independent of activity may also lead to accumulating multiple sclerosis disability.^6^

There are no published studies that explore cognitive disability in people with aggressive multiple sclerosis or with severe disability EDSS ≥ 8.0. One way to approach the cognitive research in aggressive multiple sclerosis is to evaluate those with advanced, severe disease, who are often relegated to a skilled nursing facility. The lack of cognitive data is greatly limited by the small subject pool, plus sensory and motor impediments to comprehensive neuropsychological testing. For example, the most advanced people with multiple sclerosis enrolled in the EXPAND trial had a maximum baseline EDSS score of 6.5 and were younger than real-world progressive people with multiple sclerosis.^7,8^ In many instances, people with aggressive multiple sclerosis experience substantial upper limb limitations, vision problems, dysarthria and/or severe dementia that renders them ineligible for inclusion in most studies.^9,10^ Therefore, it remains unknown whether the people with aggressive multiple sclerosis are ‘locked in a shell by their body’ with reasonably preserved cognitive abilities or whether their cognition commensurate to their physical disability.

Multiple assessment batteries have been established and validated for measurement of global and domain-specific cognitive performance in people with multiple sclerosis.^11^ In particular, majority of people with multiple sclerosis exhibit a high level of attention/cognitive processing speed impairment that is routinely measured by the Symbol Digit Modalities Test (SDMT), which is considered a gold standard in the multiple sclerosis field.^12^ That said, SDMT can be substantially influenced by visual, oculomotor and verbal impairments that are present in people with multiple sclerosis and substantially limit its utility in the aforementioned severely disabled people with multiple sclerosis.^10^ The Auditory Test of Processing Speed (ATOPS) test was recently proposed and aims at assessing attention/cognitive processing speed in a manner that uses auditory cues whereby only simple relational Boolean operations are required to be performed.^13^ This test can be utilized in severely disabled people with multiple sclerosis that would otherwise be unable to be properly assessed with a routine cognitive battery.

MRI and volumetric analysis have been increasingly utilized in the clinical care of people with multiple sclerosis.^14^ Although the MRI procedure is non-invasive, the level of physical disability in patients with severe progressive multiple sclerosis often prevents them from undergoing a typical ∼1-h-long scanning session. Therefore, very little MRI data are available from this under-researched population, which could otherwise provide greater insights into the pathological aetiology of their phenotype and extensive disability.^3^ MRI quantification of the brain and tissue-specific volumes could allow for testing of the aforementioned hypothesis that neuroaxonal damage is the driver of disability in the aggressive form of multiple sclerosis. Recent developments in artificial intelligence (AI)–based techniques enable segmentation of structural volumes on readily available images such as the fluid-attenuated inversion recovery (FLAIR) sequence,^15^ which is typically acquired as part of any multiple sclerosis imaging protocol.

Based on this background, we endeavoured to characterize cognitive function in severe progressive multiple sclerosis and understand its relationship with MRI-based measures. For this study, we developed and utilized an attention/cognitive processing speed test that would allow us to quantify cognitive function in people with multiple sclerosis with substantial motor, vision and speech difficulty. We hypothesized that people with severe progressive multiple sclerosis would have significantly greater cognitive deficits when compared with an age-, sex- and disease duration-matched counterpart (versus the locked-in hypothesis), and these changes will be associated with neurodegenerative MRI-based outcomes.

## Materials and methods

### Study participants

The cognitive analysis presented in this manuscript is part of a larger multi-stage project called Comprehensive Assessment of Severely Affected Multiple Sclerosis (CASA-MS). This prospective cross-sectional analysis utilized data from people with multiple sclerosis that were enrolled at two sites including the Boston Home (TBH), a specialized tertiary care multiple sclerosis facility in Dorchester, MA, USA, and at the Jacobs Multiple Sclerosis Center for Treatment and Research (JMSCTR), Department of Neurology, University at Buffalo, NY, USA. The study enrolled people with multiple sclerosis that were matched based on sex, ±2 years of age and ±2 years of disease duration. The inclusion criteria for the study were (i) age between 30 and 80 years old, (ii) diagnosed with multiple sclerosis based on the 2017 revision of the McDonald criteria,^16^ (iii) people with multiple sclerosis from TBH who had to have an EDSS score between 7.0 and 9.5, (iv) people with multiple sclerosis from the JMSCTR at UB (hereafter referred to as community-dwelling people with multiple sclerosis) who had to have an EDSS score between 3.0 and 6.5 and (v) having availability of a MRI scan for analysis. The exclusion criteria were (i) had experienced a relapse or had received steroids within 30 days prior to study enrolment and (ii) inability to obtain informed consent by the people with multiple sclerosis or their legal proxy. The overall study design of CASA-MS aimed at determining the demographic, clinical and MRI-based factors related to severe multiple sclerosis. By matching people with multiple sclerosis for their sex, age and the age at multiple sclerosis onset (disease duration), we aimed at isolating other variables that could explain the differences in their disability. All participants were examined by a trained and Neurostatus-certified researcher (D.J.) under the supervision of board-certified neurologist with more than 30 years of experience (B.W.-G.). People with multiple sclerosis were classified as either (i) relapsing secondary progressive multiple sclerosis, (ii) non-relapsing secondary progressive multiple sclerosis or (iii) primary progressive multiple sclerosis based on the 2013 Lublin criteria.^17^ Standardized questionnaires were additionally completed to collect information regarding the age at multiple sclerosis onset, time from symptom onset to multiple sclerosis diagnosis, years of education, body mass index (BMI), the occurrence of relapses in the previous 24 months before study participation and the use/type of disease-modifying therapy (DMT).

The study was approved by the local Institutional Review Board, and informed consent was obtained. When the participants were not physically or cognitively able to provide their own consent, a legally authorized representative consented to the study on their behalf.

### Neuropsychological examination

All neuropsychological tests were performed by a trained investigator (D.J., Z.W. and T.R.W.) under supervision of a board-certified neuropsychologist (R.H.B.B.). The Brief International Cognitive Assessment for Multiple Sclerosis was administered,^18^ with other tests emphasizing auditory processing and speech.

First, the attention/cognitive processing speed was assessed by the SDMT,^19^ the Paced Auditory Serial Addition Test—3 second (PASAT-3)^20^ and ATOPS.^13^ ATOPS, a smartphone-based test of mental speed, requires only simple relational and Boolean operations performed on auditive numeric stimuli. The validation of the test has been recently published.^13^ In particular, the test presents auditive numbers between 1 and 99 and requires examinees to make a binary oral decision as fast as possible. The ATOPS testing encompasses 4 different trials, where the first one is used as a calibration measure that determines the administrator's reaction time over 20 auditive stimuli. The following 3 test trials also present 20 stimuli. First, subjects have to respond ‘yes’ if the number is greater than 50 and ‘no’ if it is less than 50. The second and third trials present numbers, and participants have to say yes or no based on a conjunction or disjunction of odd/even and >50/<50. In order to decrease the number of multicollinear comparison, only the third trial of the ATOPS test was used in this analysis. In our validation manuscript, the third trial of ATOPS had the highest convergent validity with SDMT and highest correlation coefficient with lesion pathology in multiple sclerosis.^13^ Moreover, the ATOPS data were screened for technical errors and participant accuracy ≤50%; such cases were excluded from final group comparisons. ATOPS trial times were corrected for administrator reaction time and participant accuracy. Being non-normally distributed, these adjusted processing times underwent log transformation.

As a second and third domain, the learning and memory were assessed by the California Verbal Learning Test—2nd Edition (CVLT-II)^21^ and the visuospatial domain using the Brief Visuospatial Memory Test—Revised (BVMT-R).^22^ In both memory tests, the respective measures of immediate recall (IR), delayed recall (DR) and recognition index (RI) were quantified.

Lastly, the language domain was examined using the Controlled Oral Word Association Test (COWAT),^23^ Neuropsychological Assessment Battery (NAB) naming test and maximum repetition rate (MRR) test. The COWAT specifically quantifies letter-based and categorical verbal fluency, the NAB naming quantifies visual confrontational naming skills (identifying aphasia) and MRR assesses speech motor performance. In order to shorten the time of neuropsychological visit and due to substantial disability of people with multiple sclerosis in the skilled nursing facility, the fifth domain of executive function as outlined by the International Classification of Cognitive Disorders in Multiple Sclerosis was not assessed.^24^

### MRI acquisition and analyses

The MRI scans for all participants were retrospectively collected from their medical records and all downloaded to a central server for analysis. The minimal requirement for study participation was 1.5T or 3T MRI scan with at least 2D T2-FLAIR sequence. Additional sequences utilized in these analyses were 2D or 3D T1-weighed spin echo and high-resolution 3D T1-weighted imaging. The T2-FLAIR scans had median slice thickness of 1.2 mm (1.2 mm for community-dwelling people with multiple sclerosis and 1.8 mm for skilled nursing facility people with multiple sclerosis), whereas the T1-WI sequence had median slice thickness of 1.2 mm (1.2 and 3.0 mm for community-dwelling and skilled nursing facility people with multiple sclerosis, respectively). Detailed MRI scanner distribution for the study population was published elsewhere.^25^

All scans were batch analysed by a central neuroimaging analysis site [Buffalo Neuroimaging Analysis Center (BNAC), NY, USA] and without knowledge regarding the status of people with multiple sclerosis. The scans were transferred from each of the MRI acquisition sites using either a direct imaging transfer portal or by providing copies via compact discs. BNAC has more than 20 years of experience as a core imaging facility with both ISO 9001:2015 (quality management) and ISO 27001:2022 (information security) certifications. T2 and T1 lesion volumes (LV) were determined using an AI-based automated segmentation.^26^ All lesion segmentations were quality controlled by a trained neuroimager (D.J., M.G.D. and N.B. with combined 50 years of imaging experience) and manually adjusted as needed. T1-weighted images were pre-processed for bias field correction and T1 hypointensity filling.^27^ Segmentation of the brain volumes, including the whole brain volume (WBV), white matter volume, grey matter volume (GMV) and cortical volume (CV), was performed using the cross-sectional Structural Image Evaluation, using Normalisation, of Atrophy (SIENAX, https://fsl.fmrib.ox.ac.uk/fsl/fslwiki/SIENA, version 2.6), while deep GMV (sum volume of the thalamus, globus pallidus, putamen, caudate, hippocampus and amygdala) and thalamic volumes (TV) were calculated using FMRIB's Integrated Registration and Segmentation Tool (https://fsl.fmrib.ox.ac.uk/fsl, version 1.2).^28^ All volumes were normalized for head size using the SIENAX-derived scaling factor.

An in-house developed panel of T2-FLAIR-derived quantitative measures was processed as described elsewhere.^15^ In particular, the T2-FLAIR panel provided measures of (i) TV, using the DeepGRAI algorithm;^29^ (ii) lateral ventricular volume (LVV) using the NeuroSTREAM algorithm;^30^ (iii) salient central lesion volume;^31^ (iv) medulla oblongata volume, as a proxy of spinal cord atrophy; and (v) thalamic dysconnectivity,^32^ which reflects the degree of lesion-induced network dysconnectivity within white matter tracts connected to the thalamus. These measures have been previously validated as comparable proxy measures to the high-resolution 3D T1-WI-based counterparts.^15^

### Statistical analyses

All statistical analyses were performed using SPSS version 28.0 (IBM, Armonk, NY, USA), and the data were visualized using GraphPad Prism (San Diego, CA, USA). All data input was checked for potential inconsistencies, and 10% of data points were manually checked as well. The data distribution was determined by visual inspection of the histograms and Q–Q plots. Categorical data was compared using χ^2^ test, normally distributed parametric data using Student's *t*-test and non-parametric data using Mann–Whitney U-test. Variables with Poisson distribution (e.g. number of relapses) were compared using negative binomial regression. The effect size was determined using Cohen's *d* where 0.2–0.5, 0.5–0.8 and >0.8 were considered small, medium and large effect sizes, respectively.

The relationships between representative cognitive performance measures and the MRI-based measures were determined using Spearman's correlation and stepwise linear regression models. The models used the cognitive performance score as a dependent variable. All linear regression models were adjusted for sex, age and years of education independent covariates and all MRI-based measures in a stepwise addition independent predictor. Only significant MRI-based measures were included in the final model output. The same regression models for both community-dwelling people with multiple sclerosis and people with multiple sclerosis in the skilled nursing facility were performed. The false discovery rate was corrected using the Benjamini–Hochberg procedure. *P*-values lower than 0.05 were considered statistically significant.

## Results

### Demographic and clinical characteristics of the study population

The demographic and clinical characteristics of the study population are shown in Table 1. In total, 87 people with multiple sclerosis (43 community-dwelling people with multiple sclerosis and 44 people with multiple sclerosis at the skilled nursing facility) underwent neuropsychological testing. When compared with the community-dwelling people with multiple sclerosis, people with multiple sclerosis in the skilled nursing facility were significantly more disabled (median EDSS 8.0 versus 5.5, *P* < 0.001) and had greater percentage of progressive multiple sclerosis disease course (81.8% versus 34.9%, *P* < 0.001) and lower average BMI (25.6 versus 28.1, *P* = 0.038). Based on the study matching criteria, there were no differences in sex distribution (*P* = 0.563), age (*P* = 0.31) and disease duration (*P* = 0.253) between the groups. Albeit not statistically significant, a greater percentage of people with multiple sclerosis at the nursing facility were not on any DMT when compared with the community-dwelling people with multiple sclerosis (61.4% versus 34.9%, *P* = 0.067). There were no differences in the time between the MRI and cognitive examination between both people with multiple sclerosis groups (1.5 versus 2.6, *P* = 0.073).

**Table 1:** Demographic and clinical characteristics of the study population

Demographic and clinical characteristics	Community-dwelling pwMS (*n* = 43)	pwMS in skilled nursing facility (*n* = 44)	*P*-value
Female, *n* (%)	28 (65.1)	26 (59.1)	0.563^a^
Race, *n* (%)			
Caucasian	38 (88.4)	43 (97.7)	0.085^a^
African-American	5 (11.6)	1 (2.3)	
Disease course, *n* (%)			
Relapsing progressive	28 (65.1)	8 (18.2)	**<0.001** ^a^
Non-relapsing progressive	12 (27.9)	36 (81.8)	
Primary progressive	3 (7.0)	0 (0.0)	
Time between cognitive and MRI examination in years, median (IQR)	1.5 (2.3)	2.6 (2.4)	0.073^b^
Age in years, mean (SD)	61.3 (8.6)	63.3 (9.2)	0.31^b^
Age at onset in years, mean (SD)	33.7 (9.8)	33.3 (10.6)	0.854^b^
Time to diagnosis, mean (SD)	4.4 (7.2)	4.1 (7.0)	0.842^b^
BMI, mean (SD)	28.1 (5.6)	25.6 (5.4)	**0.038** ^b^
Education (years), mean (SD)	14.4 (2.3)	14.8 (3.0)	0.565^b^
Disease duration (years), mean (SD)	27.9 (9.9)	30.3 (9.4)	0.253^b^
Relapse in previous 24 months, mean (SD)	0.14 (0.413)	0.045 (0.211)	0.184^c^
EDSS, median (IQR) mean	5.5 (3.5–6.0)	8.0 (8.0–8.375)	**<0.001** ^d^
DMT, *n* (%)			
Interferon-β	6 (14.0)	2 (4.5)	0.067^a^
Glatiramer acetate	4 (9.3)	7 (15.9)	
Teriflunomide	5 (11.6)	4 (9.1)	
Fingolimod/siponimod	1 (2.3)	1 (2.3)	
Anti-CD20	8 (18.6)	3 (6.8)	
Natalizumab	3 (7.0)	0 (0.0)	
Others	1 (2.3)	0 (0.0)	
No therapy	15 (34.9)	27 (61.4)	

*P*-values lower than 0.05 were considered statistically significant and shown in bold. pwMS, people with multiple sclerosis; MRI, magnetic resonance imaging; SD, standard deviation; BMI, body mass index; EDSS, expanded disability status scale; IQR, interquartile range; DMT, disease-modifying treatment.

^a^χ^2^ test.

^b^Student’s *t*-test.

^c^Negative binomial regression.

^d^Mann–Whitney U-test.

### Neuropsychological test completion rate and cognitive performance

**Figure 1:**
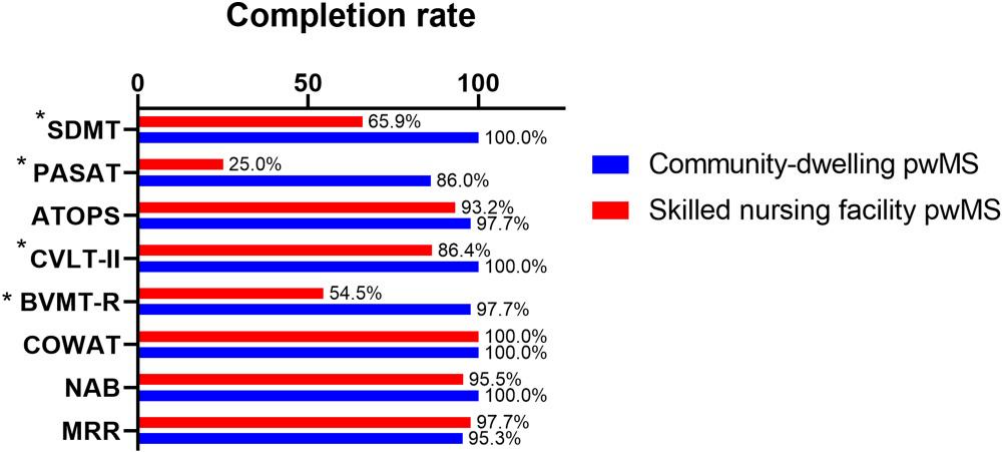
**Completion rate of representative neuropsychological tests in the study groups**. A significantly greater number of people with multiple sclerosis from the skilled nursing facility were able to have their cognitive processing speed tested using the ATOPS versus the SDMT (93.2% versus 65.9%). All measures are represented as a percentage of test completion. For example, 29 out of 44 (65.9%) people with multiple sclerosis from a skilled nursing facility completed the SDMT test. The difference between completion rates between the community-dwelling and skilled nursing facility people with multiple sclerosis is compared using a χ^2^ test. *P*-value lower than 0.05 was considered statistically significant and labelled as an asterisk at the name of the test. pwMS, people with multiple sclerosis; SDMT, Symbol Digit Modalities Test; PASAT-3, Paced Auditory Serial Addition Test—3 second; ATOPS, Auditory Test of Processing Speed; CVLT-II, California Verbal Memory Test—2nd Edition; BVMT-R, Brief Visuospatial Memory Test—Revised; COWAT, Controlled Oral Word Association Test; NAB, Neuropsychological Assessment Battery naming test; MRR, maximum repetition rate.

The successful completion rates for each neuropsychological test in both the community-dwelling and nursing facility people with multiple sclerosis are shown in Table 2 and Fig. 1. ATOPS was successfully completed by the majority of people with multiple sclerosis (97.7% by community-dwelling people with multiple sclerosis and 93.2% by the skilled nursing facility people with multiple sclerosis). Significantly greater number of people with multiple sclerosis from the nursing facility failed to complete both the SDMT (65.9% versus 100%, *P* < 0.001) and PASAT-3 tests (25% versus 86%, *P* < 0.001) when compared with their less disabled, community-dwelling counterparts. Within the community-dwelling people with multiple sclerosis, there were no differences in the completion rate of ATOPS versus SDMT (97.7% versus 100%), whereas greater number of the nursing facility people with multiple sclerosis completed ATOPS versus SDMT (93.2% versus 65.9%, *P* = 0.013).

**Table 2:** Completion rate for each of the neuropsychological tests used in the study

Test completion rate	Community-dwelling pwMS (*n* = 43)	pwMS in skilled nursing facility (*n* = 44)	*P*-value
Attention/cognitive processing speed domain			
SDMT, *n* (%)	43 (100.0)	29 (65.9)	**<0.001**
PASAT-3, *n* (%)	37 (86.0)	11 (25.0)	**<0.001**
ATOPS, *n* (%)	42 (97.7)	41 (93.2)	0.317
Learning and memory domain			
CVLT-II, *n* (%)	43 (100.0)	38 (86.4)	**0.012**
BVMT-R, *n* (%)	42 (97.7)	24 (54.5)	**<0.001**
Language domain			
COWAT, *n* (%)	43 (100.0)	44 (100.0)	1.000
NAB, *n* (%)	43 (100.0)	42 (95.5)	0.157
MRR, *n* (%)	41 (95.3)	43 (97.7)	0.543

All comparisons were performed using χ^2^ test. *P*-values lower than 0.05 were considered statistically significant and shown in bold. pwMS, people with multiple sclerosis; SDMT, Symbol Digit Modalities Test; PASAT-3, Paced Auditory Serial Addition Test—3 second; ATOPS, Auditory Test of Processing Speed; DSB, Digit Span Backwards; CVLT-II, California Verbal Memory Test—2nd Edition; BVMT-R, Brief Visuospatial Memory Test—Revised; COWAT, Controlled Oral Word Association Test; NAB, Neuropsychological Assessment Battery naming test; MRR, maximum repetition rate.

A significantly greater proportion of people with multiple sclerosis from the skilled nursing facility also failed to complete both the verbal and visuospatial learning and memory tests (86.4% versus 100%, *P* = 0.012 for CVLT-II and 54.5% versus 97.7%, *P* < 0.001 for BVMT-R). There were no differences in the completion rate within the three tests that examine the language domain (COWAT, NAB and MRR).

**Figure 2:**
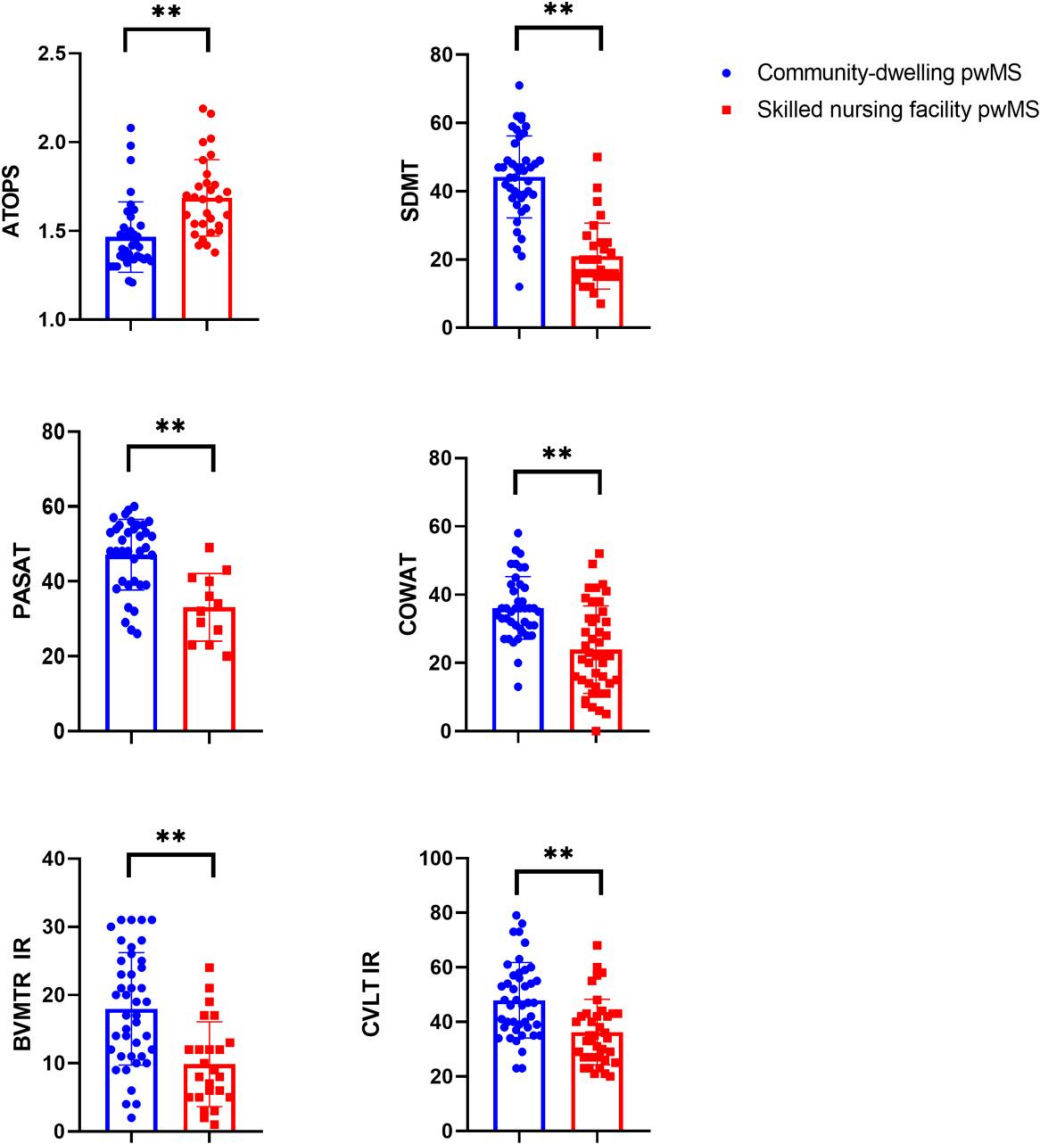
**Box plot demonstrating the differences in cognitive performance between the study groups.** Statistically significant differences are labelled using **P < 0.001. Student's *t*-test was used for all comparisons. There were significant differences between the skilled nursing facility and community-dwelling people with multiple sclerosis on all cognitive measures including cognitive processing speed (ATOPS, SDMT, PASAT-3), verbal and visuospatial learning and memory (CVLT-II and BVMT-R) and language (COWAT). pwMS, people with multiple sclerosis; SDMT, Symbol Digit Modalities Test; PASAT-3, Paced Auditory Serial Addition Test—3 second; ATOPS, Auditory Test of Processing Speed; CVLT-II, California Verbal Memory Test—2nd Edition; IR, immediate recall; BVMT-R, Brief Visuospatial Memory Test—Revised; COWAT, Controlled Oral Word Association Test.

The cognitive performance and differences between the community-dwelling and skilled nursing facility people with multiple sclerosis groups are shown in Table 3. In general, nursing facility subjects had significantly worse performance throughout the entire neuropsychological battery and in all of the cognitive domains, except for the NAB test (*P* = 0.106). Based on the effect size, the greatest difference between the people with multiple sclerosis groups was in SDMT (21 versus 44.2, *P* < 0.001, Cohen's *d* = 2.13), followed by PASAT-3 (32.2 versus 47, *P* < 0.001, Cohen's *d* = 1.626), BVMT-R IR (9.9 versus 18, *P* < 0.001, Cohen's *d* = 1.111) and ATOPS (1.7 versus 1.5, *P* < 0.001, Cohen's *d* = 1.069). The visual representation of the differences in cognitive performance between the groups is shown in Fig. 2.

**Table 3:** Differences in cognitive performance between the study groups

Cognitive performance	Community-dwelling pwMS	pwMS in skilled nursing facility	*P*-value	Cohen's *d*
Attention/cognitive processing speed domain				
SDMT	44.2 (12)	21 (9.7)	**<0.001**	2.13
PASAT-3	47 (9.3)	32.2 (8.9)	**<0.001**	1.626
ATOPS	1.5 (0.2)	1.7 (0.2)	**<0.001**	1.069
Learning and memory domain				
CVLT-II IR	48 (13.8)	36.3 (12)	**<0.001**	0.903
CVLT-II DR	9.6 (4)	6.4 (4.1)	**0.001**	0.784
CVLT-II RI	3 (0.8)	2.2 (0.9)	**<0.001**	1.006
BVMT-R IR	18 (8.2)	9.9 (6.2)	**<0.001**	1.111
BVMT-R DR	7 (3.3)	4 (2.5)	**<0.001**	1.02
BVMT-R RI	5.4 (0.9)	4.4 (1.6)	**0.007**	0.768
Language domain				
COWAT	35.1 (10.1)	23.9 (12.8)	**<0.001**	0.97
NAB	29.6 (2.9)	28.5 (3.2)	0.106	0.354
MRR	1.38 (0.74)	0.98 (0.51)	**0.005**	0.623

All tests are shown as mean (SD) unless otherwise specified. All comparisons were performed using Student's *t*-test. *P*-values lower than 0.05 were considered statistically significant and shown in bold. pwMS, people with multiple sclerosis; SDMT, Symbol Digit Modalities Test; PASAT-3, Paced Auditory Serial Addition Test—3 second; ATOPS, Auditory Test of Processing Speed; CVLT-II, California Verbal Memory Test—2nd Edition; IR, immediate recall; DR, delayed recall; RI, recognition index; BVMT-R, Brief Visuospatial Memory Test—Revised; COWAT, Controlled Oral Word Association Test; NAB, Neuropsychological Assessment Battery naming test; MRR, maximum repetition rate.

We performed an additional analysis to compare the ATOPS scores in people that failed SDMT (unable to perform the test) and compared it to their matched community-dwelling people with multiple sclerosis. The same numerical group differences in ATOPS from the total sample size were seen in the subset of people with multiple sclerosis that failed the traditional cognitive tests. In particular, the people with multiple sclerosis from the skilled nursing facility were numerically worse in ATOPS when compared with the community-dwelling people with multiple sclerosis (1.8 versus 1.6, *P* = 0.09, Cohen's *d* = 0.81).

In order to parcel out the effect of dysarthria and its effect on the neuropsychological performance, an additional comparison between the study sites was performed and adjusted by the MRR score. In the total study population, worse MRR score (greater motor speech impairment) was significantly associated with worse SDMT score (*r* = 0.29, *P* = 0.015), PASAT-3 score (*r* = 0.31, *P* = 0.005) and COWAT (*r* = 0.306, *P* = 0.005). Within the attention/cognitive processing speed domain, ATOPS was not associated with the motor speech impairment level.

After adjusting for the MRR score, the differences between people with multiple sclerosis groups remained significant for ATOPS (η^2^ = 0.198, *P* < 0.001), PASAT-3 (η^2^ = 0.276, *P* < 0.001) and SDMT (η^2^ = 0. 481, *P* < 0.001).

### Relationship of cognitive performance and MRI-based measures

The relationship between the cognitive performance and the MRI measures in the total population is shown in Table 4. After adjusting for sex, age and years of education, poorer attention/cognitive processing speed assessed by SDMT was additionally and significantly explained by lower TV (*R*^2^ increase from 0.05 to 0.411, standardized β = 0.612, *P* < 0.001) and lower CV (*R*^2^ increase from 0.411 to 0.524, standardized β = 0.415, *P* = 0.001). Overall, 52.4% of the SDMT variance was explained by demographic and MRI-based measures. The WBV association did not survive multiple comparison correction (*P* = 0.047). Poorer attention/cognitive processing speed assessed by ATOPS was significantly associated with lower TV (*R*^2^ increase from 0.088 to 0.28, standardized β = −0.443, *P* < 0.002).

**Table 4:** Relationship between cognitive performance and MRI measures

	Predictor	Standardized β	*t*	*P*-value
SDMT				
*R*^2^	Sex	0.254	2.121	**0.040**
	Age	−0.185	−1.583	0.120
0.050	Education in years	0.031	0.292	0.772
0.411	TV	0.612	4.123	**<0.001**
0.524	CV	0.415	3.502	**0.001**
0.565	WBV	−0.269	−2.047	0.047^a^
ATOPS				
*R*^2^	Sex	−0.127	−0.927	0.359
	Age	0.338	2.315	**0.026**
0.088	Education in years	−0.262	−1.841	0.073
0.280	TV	−0.443	−3.346	**0.002**
CVLT-II IR				
*R*^2^	Sex	0.203	1.848	0.070
	Age	0.042	0.366	0.716
0.123	Education in years	0.204	1.788	0.080
0.346	CV	0.440	4.009	**<0.001**
0.41	T1-LV	−0.257	−2.377	**0.021**
BVMT-R IR				
*R*^2^	Sex	0.100	0.697	0.489
	Age	−0.199	−1.306	0.198
0.010	Education in years	0.012	0.083	0.935
0.167	TV	0.419	2.914	**0.006**
COWAT				
*R*^2^	Sex	0.232	2.097	**0.041**
	Age	0.152	1.335	0.187
0.107	Education in years	0.112	1.044	0.301
0.396	TV	0.429	3.503	**0.001**
0.445	CV	0.260	2.208	**0.031**

Linear stepwise regression models were used with sex, age and years of education as covariates and MRI outcomes as independent predictors of cognitive performance (dependent variable). *P*-values lower than 0.05 were considered statistically significant and shown in bold. SDMT, Symbol Digit Modalities Test; ATOPS, Auditory Test of Processing Speed; CVLT-II, California Verbal Memory Test—2nd Edition; IR, immediate recall; BVMT-R, Brief Visuospatial Memory Test—Revised; COWAT, Controlled Oral Word Association Test; WBV, whole brain volume; CV, cortical volume; TV, thalamic volume; LV, lesion volume.

^a^Not surviving Benjamini–Hochberg correction for false discovery rate.

The interaction analysis demonstrated a significant group effect on the SDMT performance (group × predicted SDMT values as predictor, F = 16.7, *P* < 0.001). Similarly, the interaction with the group had a significant effect on the ATOPS performance (group × predicted ATOPS values as predictor, F = 6.0, *P* = 0.005). The different slopes for the group and their interaction for both SDMT and ATOSP are shown in Supplementary Fig. 1.

**Figure 3:**
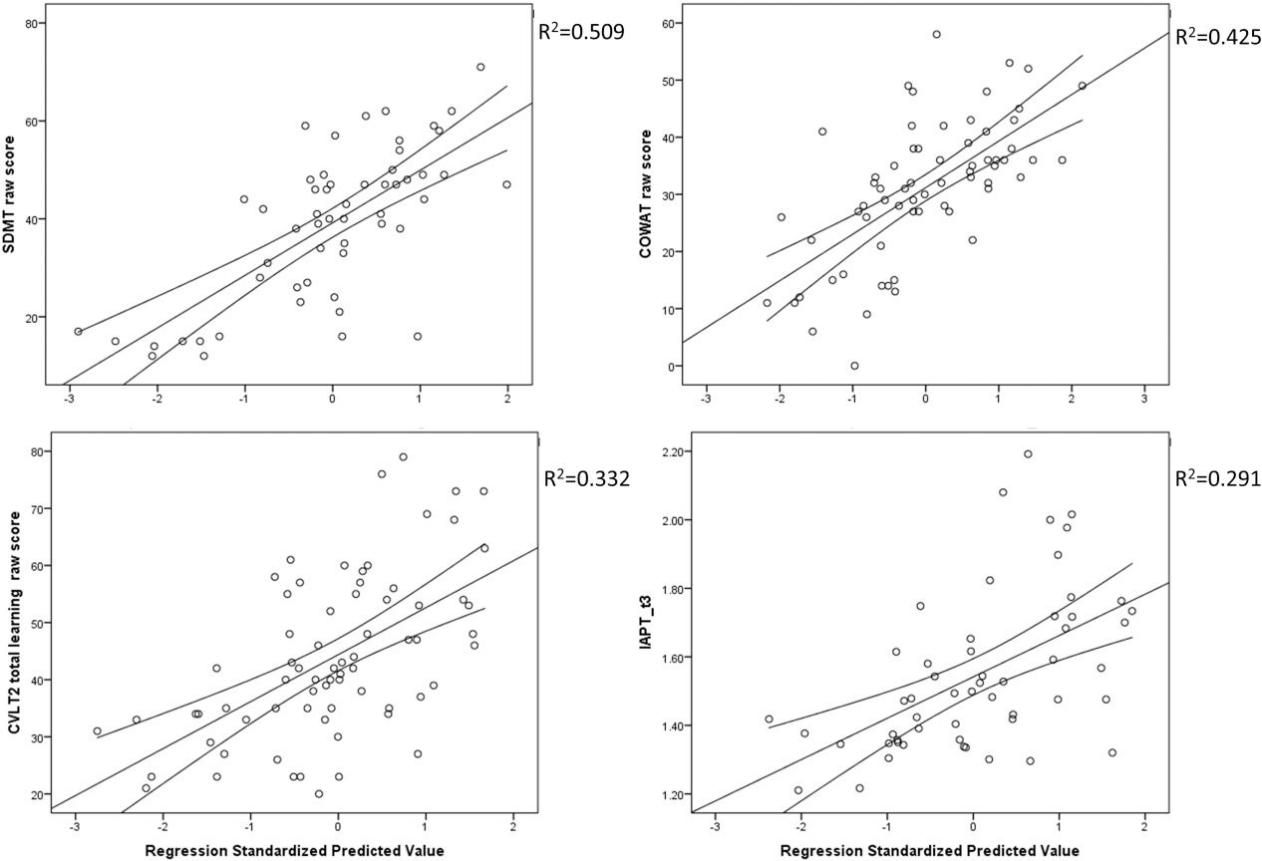
**Scatter plot demonstrating the best fit from the MRI-based linear regression models for SDMT (top left), COWAT (top right), CVLT-II IR (bottom left) and ATOPS (bottom right)**. Linear stepwise regression models were used with sex, age and years of education as covariates and MRI outcomes as independent predictors of cognitive performance (dependent variable). The significant predictors for both SDMT and COWAT were TV and CV. Significant predictors of ATOPS were only TV, whereas for CVLT-II, they were T1 LV and CV. SDMT, Symbol Digit Modalities Test; APT, accuracy-adjusted processing time; CVLT2, California Verbal Learning Test—2nd Edition; COWAT, Controlled Oral Word Association Test; IR, immediate recall.

Poorer verbal learning and memory performance assessed by CVLT-II was significantly associated with lower CV (*R*^2^ change from 0.123 to 0.346, standardized β = 0.44, *P* < 0.001) and higher T1-LV (*R*^2^ change from 0.346 to 0.41, standardized β = −0.257, *P* = 0.021). In total, 41% of the CVLT-II IR variance was explained by demographic and MRI-based measures. The visuospatial verbal and learning performance assessed by BVMT-R was significantly associated with TV (*R*^2^ change from 0.01 to 0.167, standardized β = 0.419, *P* < 0.006). Lastly, verbal fluency assessed by COWAT was significantly associated with TV (*R*^2^ change from 0.107 to 0.396, standardized β = 0.429, *P* = 0.001) and CV (*R*^2^ change from 0.396 to 0.445, standardized β = 0.26, *P* = 0.031). In total, 44.5% of the verbal fluency variance was explained by demographic and MRI-based measures. The relationships between the significant predictors and the cognitive performance in the processing speed domain (SDMT and ATOPS), verbal learning and memory (CVLT-II) and verbal fluency (COWAT) are shown in Fig. 3.

MRI-based regression analyses were performed individually for the community-dwelling people with multiple sclerosis and the people with multiple sclerosis in the skilled nursing facility and are shown in Supplementary Table 1. In the community-dwelling people with multiple sclerosis, the SDMT variance was additionally explained by CV (*R*^2^ change from 0.006 to 0.352, standardized β = 0.597, *P* < 0.001), the additional variance in ATOPS by T2-LV (*R*^2^ change from 0.129 to 0.34, standardized β = 0.463, *P* = 0.007), CVLT-II IR by CV (*R*^2^ change from 0.06 to 0.332, standardized β = 0.529, *P* = 0.001) and also COWAT by CV (*R*^2^ change from 0.143 to 0.37, standardized β = 0.484, *P* = 0.001). Lines represent the best fit and 95% confidence intervals.

In the people with multiple sclerosis at the skilled nursing facility, the CVLT-II IR variance was additionally explained by the TV (*R*^2^ change from 0.06 to 0.332, standardized β = 0.631, *P* = 0.007), and the additional BVMT-IR variance was explained by both LVV (*R*^2^ change from 0.159 to 0.669, standardized β = 0.652, *P* = 0.004) and TV (*R*^2^ change from 0.669 to 0.892, standardized β = −0.500, *P* = 0.013).

## Discussion

The findings from this study are multifold. First, the development and utilization of a specifically designed auditory cognitive test such as ATOPS, which can be performed by severe progressive multiple sclerosis participants, allow collection of quantitative data from a larger, previously unexaminable population. Moreover, the rate of routine cognitive test completion can provide additional information regarding the extent of cognitive impairment in multiple sclerosis. Interpretation of cognitive performance data that exclude patients with severe impairment of vision and upper extremity dexterity underestimates the true degree and frequency of impairment. If investigators include such patients, the correlation MRI indicators of neurodegenerative pathology, especially involving the cortex and deep gray matter, may increase. Our data suggest that there are significant differences in the cognition–MRI relationship between the community-dwelling and skilled nursing facility people with multiple sclerosis. The effects of vision and motor function notwithstanding, speech is also a requirement for many cognitive tests. The MRR was significantly associated with SDMT, PASAT-3 and COWAT, but not ATOPS, which requires only a single syllable yes/no response. Future studies may need to further adjust for this confounding factor and consider motor speech impairment when choosing the cognitive test battery.

The differences in completion rate, performance on the cognitive test and the extent to which the test captures the cognitive process warrant further exploration. While we expected that the higher completion on ATOPS (acquiring quantitative data from the most disabled people with multiple sclerosis) would result in greater differences between community-dwelling and skilled nursing facility people with multiple sclerosis, the SDMT still demonstrated greater differentiation between the groups. This finding could potentially be explained by (i) the performance on SDMT that captures greater cognitive network including memory and learning when compared with processing speed–specific ATOPS,^33,34^ (ii) visual impairments that may compound the SDMT performance and increase the gap between the multiple sclerosis groups with differential severity and (iii) ATOPS that may have a ceiling effect that underestimates the performance of the community-dwelling people with multiple sclerosis group. Construct validity research will be useful to see whether SDMT and ATOPS have similar factor loadings for latent constructs such as processing speed. Our study further corroborates the stand-alone utility of SDMT as a gold standard test for attention/cognitive processing speed that demonstrated highest ability to separate the two people with multiple sclerosis groups. ATOPS was not designed to replace SDMT, but to be utilized in people with multiple sclerosis with visual impairment, significant cognitive decline and severe disability.

Based on our literature search, few studies have reported cognitive performance in people with multiple sclerosis with severe disability. For example, a study of 43 moderate to severely disabled people with multiple sclerosis reported mean SDMT performance of 32 correct responses and mean Rey Auditory Verbal Learning Test (similar to CVLT-II test) of 39.2 words.^35^ While our people with multiple sclerosis in the skilled nursing facility were substantially more disabled when compared with the previous publication (median EDSS 8.0 versus 6.5), they performed similarly in both the SDMT and verbal memory tests.^35^ The better cognitive performance can be potentially contributed to the structured rehabilitative programme present at the facility, peer-based people with multiple sclerosis engagement and overall better care within a specialized multiple sclerosis tertiary care centre. That said, the majority of studies with people with multiple sclerosis > 8.0 are motor rehabilitative in nature and generally exclude participants with severe cognitive impairments.^36^ Lastly, the PASAT-3 effect size should be interpreted with caution, given that only 25% of the people with multiple sclerosis in the skilled nursing facility completed this test. Moreover, PASAT-3 has been gradually phased out in favour of SDMT in many multiple sclerosis guidelines and regulatory studies due to its greater practice effect along with lower clarity and engagement.^37^

The MRI analysis from both people with multiple sclerosis groups corroborated the anatomical–cognitive relationships that are previously established in the overall people with multiple sclerosis population and published in the literature.^38^ In particular, neurodegenerative changes resulting in smaller TV and CV have been recognized as one of the main drivers of cognitive decline in people with multiple sclerosis, with a particularly consistent relationship between DGM changes and performance within the attention/cognitive processing speed domain.^11^ The ‘discrepancies’ in relationships between MRI measures with ATOPS versus SDMT may provide additional information regarding their anatomical–cognitive constructs. The greater cognitive network capture by SDMT can also be appreciated not only through its significant relationship with processing speed–specific TV but also with significant cortical determinant. Such relationship was not present for ATOPS. Moreover, recent accumulating evidence suggests that verbal fluency is an emerging impairment that may be specific to the older people with multiple sclerosis population and cortical atrophy, when the semantic or category-based cues are employed.^39,40^ Over the multiple sclerosis disease course, the general atrophy pattern gradually transitions from the central brain region (thalamic atrophy and lateral ventricular expansion) in the relapsing forms of multiple sclerosis to cortical neurodegeneration (bilateral temporal pole and entorhinal cortex) in people with multiple sclerosis with progressive phenotype.^41^ As cortical atrophy accelerates, cognitive worsening does as well.^42^ Due to the limited sample size, the site-specific findings regarding the relationship between the MRI measures and cognitive performance should be interpreted with caution. Since a greater percentage of community-dwelling people with multiple sclerosis had full data availability (all MRI measures and successful completion of the cognitive tests), the overall MRI relationship analysis was driven by this population. In a recently reported study from this cohort, we were able to demonstrate that the people with multiple sclerosis with severe progressive multiple sclerosis from the skilled nursing facility had significantly greater grey matter changes, as shown by both CV and TV reductions.^43^ Another potential explanation of the greater cognition–TV associations in the community-dwelling versus skilled nursing facility people with multiple sclerosis is the floor effect of DGM atrophy. In most severe cases of multiple sclerosis, the DGM (and the thalamus as the main structure) may have already atrophied to an extent at which further volumetric loss is minimal. Recent staging efforts have demonstrated that the amount of disease-based atrophy in the thalamus significantly reduces over time, with age being the highest contributor to volume decrease in older multiple sclerosis populations.^44^

The study does have several limitations that should be highlighted. The MRI exams utilized in this study were retrospectively collected and were not harmonized. This limitation could provide additional heterogeneity within the MRI data and the correlations with the cognitive performance. The greater variability of MRI parameters within the skilled nursing facility people with multiple sclerosis may be biasing the findings and provide an explanation for their lower MRI–cognitive associations, particularly in the cognitive processing speed domain. On the other hand, the clinical validity of the T2-FLAIR-based measures used in this study has been previously shown as fairly resistant to MRI hardware and software changes.^45,46^ We believe that having fully harmonized MRI study will only further increase the strength of the relationships that were shown in this analysis. Another potential study limitation is the relatively small sample size and the lack of a healthy control group. That said, the number of people with multiple sclerosis with severe progressive multiple sclerosis is intrinsically limited. In this particular study, we were able to successfully enrol up to two-thirds of all multiple sclerosis residents of the skilled nursing facility. Although relatively small, it is important to mention that our study may also be impacted by a selection bias. We attempted at recruiting all people with multiple sclerosis at the skilled nursing facility; however, few people with multiple sclerosis had such a severe clinical and cognitive disability that prevented any meaningful study-based interaction. These people with multiple sclerosis were pre-screened and deemed non-eligible by the trained nursing facility staff and the study investigators. That said, only few other similarly specialized facilities exist in the USA, and we were able to maximize the number of severely disabled people with multiple sclerosis recruited in the study. A planned future study will incorporate prospective MRI acquisition and cognitive assessment, which aims at determining and comparing the rate of cognitive decline in the people with multiple sclerosis with severe progressive multiple sclerosis and its relationship with MRI changes. Lastly, the retrospective nature of the MRI data limited our current analysis to only sequences that are routinely available. The CASA-MS project has multiple stages in which we aim at second, longitudinal scanning session where all people with multiple sclerosis will be imaged using the same harmonized protocol on both 3T and 7T scanners. During this second stage, we aim at implementing more advanced sequences such as diffusion-based MRI and functional MRI (fMRI) outcomes that will further enable us to explore the cognition–radiological relationships in the severe multiple sclerosis population. Moreover, the fMRI scans will also provide information regarding the potential benefit of specialized rehabilitative cognitive programmes that are being implemented at skilled nursing facilities such as TBH.

In conclusion, specifically developed attention/cognitive processing speed tests such as ATOPS are instrumental in capturing the cognitive performance of severe progressive multiple sclerosis patients who would otherwise be relegated as untestable. Although the severely disabled people with multiple sclerosis have significantly worse cognitive performance when compared with their matched counterparts, the cognitive deficits follow the expected limitations within the attention/cognitive processing speed domain, verbal/visual learning and memory and verbal fluency. These findings suggest multiple sclerosis–specific dementia in the most severe cases of multiple sclerosis. The extent of cognitive limitation in the most severe people with multiple sclerosis is associated with greater neurodegenerative pathology as measured by lower cortical and thalamic volumes. While our findings suggest diverging relationships between cognitive performance and MRI outcomes within the severe multiple sclerosis phenotype when compared with community-dwelling people with multiple sclerosis, studies with greater sample sizes are needed to further corroborate this hypothesis.

## Supplementary Material

fcae226_Supplementary_Data

## Data Availability

The data that support the findings of this study are available from the corresponding author, upon reasonable request.
