# Supplementary material for: Cognitive function in severe progressive multiple sclerosis
Source: Brain Commun. 2024 Jul 2;6(4):fcae226. doi: 10.1093/braincomms/fcae226 (PMC11250210; doi:10.1093/braincomms/fcae226)
Supplement: fcae226_Supplementary_Data [file fcae226_supplementary_data.zip › Supplementary Table 1.docx]

**Supplementary Table 1.** Relationship between cognitive performance and MRI measures separately in the community-dwelling pwMS.

| **Community-dwelling pwMS** | | | | |
| --- | --- | --- | --- | --- |
| **SDMT** | **Predictor** | **Standardized β** | **t** | **p-value** |
| R^2^ | Sex | -0.099 | -0.700 | 0.488 |
|  | Age | 0.004 | 0.031 | 0.975 |
| 0.006 | Education in years | 0.154 | 1.076 | 0.289 |
| 0.352 | CV | 0.597 | 4.257 | **<0.001** |
| **ATOPS** | **Predictor** | **Standardized β** | **t** | **p-value** |
| R^2^ | Sex | 0.125 | 0.753 | 0.458 |
|  | Age | 0.304 | 1.782 | 0.086 |
| 0.129 | Education in years | -0.259 | -1.588 | 0.124 |
| 0.340 | T2-LV | 0.463 | 2.939 | **0.007** |
| **CVLT-II IR** | **Predictor** | **Standardized β** | **t** | **p-value** |
| R^2^ | Sex | 0.113 | 0.784 | 0.439 |
|  | Age | 0.028 | 0.191 | 0.849 |
| 0.060 | Education in years | 0.205 | 1.412 | 0.167 |
| 0.332 | CV | 0.529 | 3.718 | **0.001** |
| **COWAT** | **Predictor** | **Standardized β** | **t** | **p-value** |
| R^2^ | Sex | -0.024 | -0.170 | 0.866 |
|  | Age | 0.375 | 2.644 | **0.012** |
| 0.143 | Education in years | 0.065 | 0.464 | 0.646 |
| 0.370 | CV | 0.484 | 3.506 | **0.001** |
| **Skilled nursing facility pwMS** | | | | |
| **CVLT-II IR** | **Predictor** | **Standardized β** | **t** | **p-value** |
| R^2^ | Sex | 0.553 | 2.818 | .014 |
|  | Age | -0.283 | -1.225 | 0.241 |
| 0.060 | Education in years | 0.622 | 2.702 | **0.017** |
| 0.332 | TV | 0.631 | 3.124 | **0.007** |
| **BVMT-R IR** | **Predictor** | **Standardized β** | **t** | **p-value** |
| R^2^ | Sex | -0.204 | -1.373 | 0.219 |
|  | Age | -0.549 | -2.732 | **0.034** |
| 0.159 | Education in years | 0.567 | 2.864 | **0.029** |
| 0.669 | LVV | 0.652 | 4.533 | **0.004** |
| 0.892 | TV | -0.500 | -3.514 | **0.013** |

**Legend:** pwMS – people with multiple sclerosis, SDMT – Symbol Digit Modalities Test, ATOPS – Auditory Test of Processing Speed, CVLT-II – California Verbal Memory Test – 2nd Edition, IR – immediate recall, BVMT-R – Brief Visuospatial Memory Test – Revised, COWAT – Controlled Oral Word Association Test, LV – lesion volume, CV – cortical volume, LVV – lateral ventricular volume, TV – thalamic volume.

Linear step-wise regression models were used with sex, age and years of education as covariates and MRI outcomes as independent predictors of cognitive performance (dependent variable). P-values lower than 0.05 were considered statistically significant and shown in bold.
