# Supplementary material for: Cognitive function in severe progressive multiple sclerosis
Source: Brain Commun. 2024 Jul 2;6(4):fcae226. doi: 10.1093/braincomms/fcae226 (PMC11250210; doi:10.1093/braincomms/fcae226)
Supplement: fcae226_Supplementary_Data [file fcae226_supplementary_data.zip › Supplementary_Figure_Legend.docx]

**Supplementary Figure 1.** The differences in cognitive-MRI relationships between the pwMS from the community-dwelling settings and skilled nursing facility.

**Legend:** pwMS – people with multiple sclerosis, SDMT – Symbol Digit Modalities Test, ATOPS – Auditory Test of Processing Speed.

Linear step-wise regression models were used with sex, age and years of education as covariates and MRI outcomes as independent predictors of cognitive performance (dependent variable).
