# Supplementary figures and images for: Cognitive function in severe progressive multiple sclerosis
Source: Brain Commun. 2024 Jul 2;6(4):fcae226. doi: 10.1093/braincomms/fcae226 (PMC11250210; doi:10.1093/braincomms/fcae226)

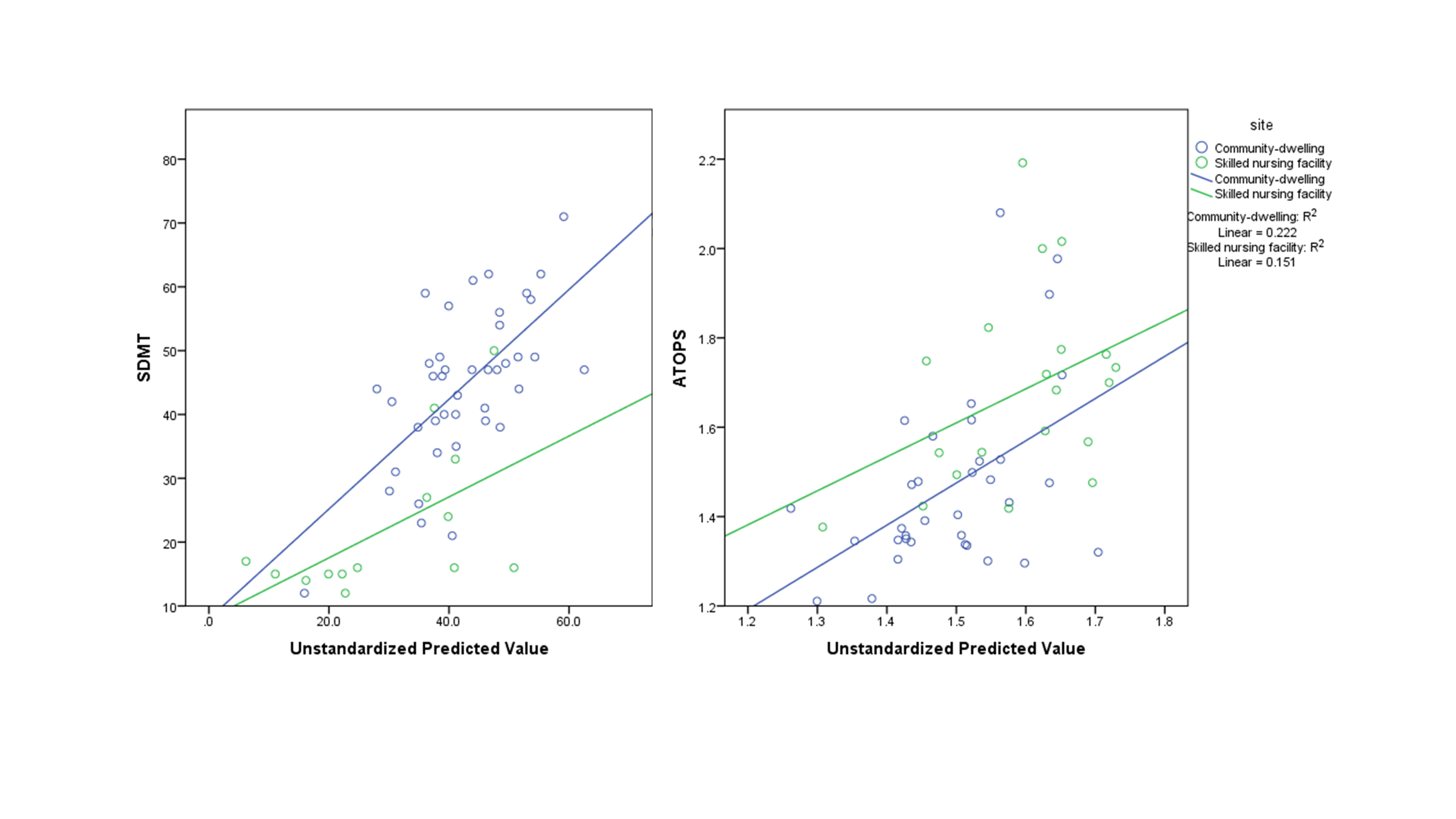

Supplement: fcae226_Supplementary_Data [file fcae226_supplementary_data.zip › Supplementary_Figure.tif]
